# Characterization of Thermo-Mechanical and Fracture Behaviors of Thermoplastic Polymers

**DOI:** 10.3390/ma7010375

**Published:** 2014-01-13

**Authors:** Elhem Ghorbel, Ismail Hadriche, Giuseppe Casalino, Neila Masmoudi

**Affiliations:** 1 Laboratory of Mechanics and Materials of the Civil Engineering (L2MGC-EA4114), University of Cergy Pontoise, 5 mail Gay-Lussac, Neuville sur Oise, Cergy Pontoise Cedex 95031, France; E-Mails: elhem.ghorbel@u-cergy.fr (E.G.); ismail_hadriche@yahoo.fr (I.H.); 2 Department of Mechanics Management and Mathematics (DMMM), Politecnico di Bari, Viale Japigia, 182, Bari 70126, Italy; 3 Electromechanical Systems Laboratory (LASEM-LR99ES36), National Engineering School of Sfax, University of Sfax, Cité El Habib BP29, Sfax 3052, Tunisia; E-Mail: neila.masmoudikhabou@enis.rnu.tn

**Keywords:** polymeric materials, torsion test, infrared camera, self-heating, thermo-mechanical behavior

## Abstract

In this paper the effects of the strain rate on the inelastic behavior and the self-heating under load conditions are presented for polymeric materials, such as polymethyl methacrylate (PMMA), polycarbonate (PC), and polyamide (PA66). By a torsion test, it was established that the shear yield stress behavior of PMMA, PC, and PA66 is well-described by the Ree-Eyring theory in the range of the considered strain rates. During the investigation, the surface temperature was monitored using an infrared camera. The heat release appeared at the early stage of the deformation and increased with the strain and strain rate. This suggested that the external work of deformation was dissipated into heat so the torsion tests could not be considered isothermal. Eventually, the effect of the strain rate on the failure modes was analyzed by scanning electron microscopy.

## 1. Introduction

The development of innovative products made of plastic materials represents a great challenge when it comes to the material selection. The choice of a suitable material for a given application is influenced by the knowledge of its behavior.

Therefore, the identification of the equations of the material behavior is highly desirable and it is generally based on experimental trials. Unfortunately, the equations depend on a great number of parameters that must be identified using a limited number of experiments. Several authors have proposed constitutive laws that accurately describe the behavior under tension and compression but overestimate the shear strength [1,2].

After the results of some experimental investigations, this can be explained by a change in the rigidity of the polymeric materials during the deformation due to a temperature change [3,4]. In fact, the temperature rise influences the mechanical behavior of the material during torsion deformation, as well as the geometry and the microstructure during welding, which calls for the inclusion of the temperature in the constitutive thermo-mechanical model of the material [5,6].

The main aim of this study is to assess the temperature modification that occurs during torsion deformation for several amorphous and semi-crystalline thermoplastic materials. The heat release appeared at the early stage of the deformation, and increased with the train and strain rate. The experiments were performed at different strain rates, using polymethyl methacrylate (PMMA), polycarbonate (PC), and polyamide (PA66) thermoplastic materials.

The mechanical tests were performed at various strain rates and room temperature. The thermographic images were recorded using an infrared camera (Fluke, Everett, WA, USA). Thermography is a measurement technique, which provides an image of the distribution of the temperature on the surface of the examined object, and allows controlling the thermal gradient without any contact [7–9].

Moreover, the failure mechanisms of the thermoplastic polymers were studied by scanning electron microscopy (SEM) (Leica, Heerbrugg St. Gallen, Switzerland).

Eventually, this paper contributes to the understanding of the relationship between deformation mechanisms and self-heating for thermoplastic polymers, which is of great interest in technological applications [10].

## 2. Materials and Experimental Methods

### 2.1. Fourier Transform Infrared Spectroscopy (FTIR) Measurement

Three industrial thermoplastics are used: polymethyl methacrylate (PMMA), polycarbonate (PC), and polyamide (PA66).

Fourier transform infrared spectroscopy (FTIR) measurements are performed in order to identify the polymers under investigation. The test was conducted using a FTIR TENSOR 27 (Bruker Optics GmbH, Wien, Austria) spectrophotometer in the range of 400–4000 cm^−1^ at a spectral resolution of 4 cm^−1^.

**Figure 1.**
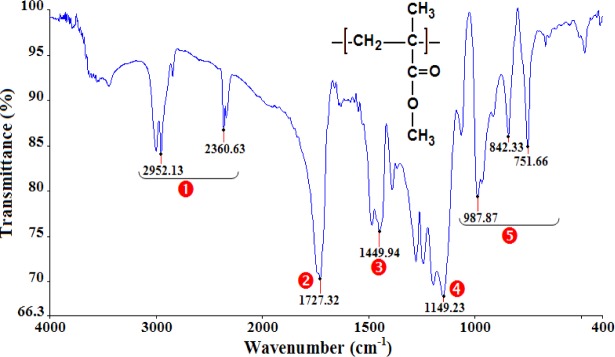
Infrared spectra of PMMA.

**Figure 2.**
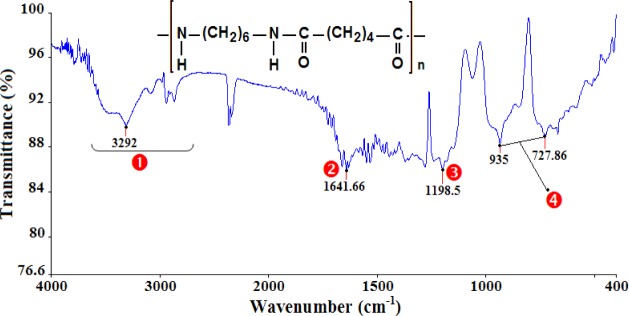
Infrared spectra of PA66.

**Figure 3.**
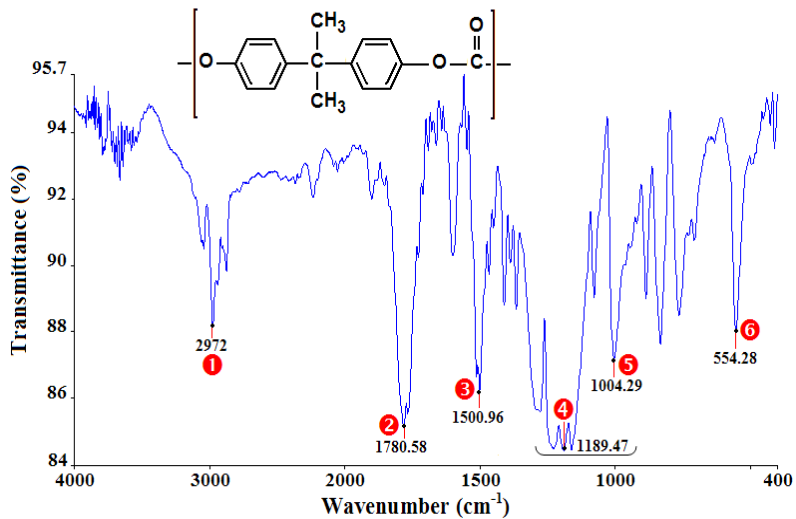
Infrared spectra of PC.

The analysis of the FTIR spectra and the assignments of the peaks (Table 1) confirm that the polymers under study are PMMA (Figure 1), PA66 (Figure 2), and PC (Figure 3).

### 2.2. Differential Scanning Calorimetric (DSC) Measurement

Differential scanning calorimetric (DSC) tests were realized to determine the physical properties of these polymers. The DSC Q100 equipment (TA Instruments, New Castle, DE, USA) with a heating rate of 10 °C/min with 25 mL/min Helium protection was used. The weight fraction crystallinity was assessed by means of Equation (1):

$$X_{c}=\frac{\Delta H_{f}}{\Delta H_{\infty }}$$ (1)

where ∆*H_f_* is the enthalpy of fusion of samples and ∆*H_∞_* is the extrapolated value of the enthalpy, which corresponds to the 100% fusion of a perfect infinite crystalline sample. For PA66, values of 188 J/g and 47.7 J/g were chosen for ∆*H_∞_* and ∆*H_f_*, respectively [11–14].

The glass transition temperature *T_g_*, the melting temperature *T*_f_, and the weight fraction crystallinity obtained using this experimental technique is shown in Table 2.

### 2.3. Mechanical Testing and Scanning Electron Microscopy

**Figure 4.**
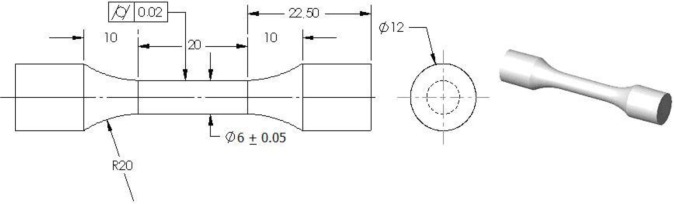
Dimensions (in mm) of the torsion test specimen.

Torsion tests were conducted on cylindrical samples, which were machined and polished from extruded bars (Figure 4). A “DELTALAB EM400” torsion testing machine (Deltalab, Carcassonne, France), equipped with strain gauges torque meters for a maximum measuring value of 20 Nm, was used. Infrared thermography was realized using a thermal camera, FLUKE “ThermoView Ti30” (Fluke, Everett, WA, USA). The objective was to quantify the change in temperature, which occurs during torsion deformation for amorphous and semi-crystalline thermoplastic, and its effect on the yielding and fractography behaviors.

This technique requires calibrating its radiant energy, which requires the emissivity “ζ” for each polymer. ζ = 0.95 can be used for all the tested polymers. The measured temperature is close to the actual one, with an accuracy of ±2% and a resolution of 0.1 °C. Several images were captured during each test. The analysis of these thermographs was done with the “InsideIR-FLUKE” software (Fluke, Everett, WA, USA).

Torsion tests were performed at room temperature (*T* = 23 °C) in order to investigate the effects of strain rates on the yielding, the mechanical characteristics, and self-heating of glassy and semi-crystalline polymers. The fracture surfaces to be characterized by scanning electron microscopy (SEM) were treated with metallization with gold, with “POLARON Sputter Coater SC502” (Gala Instrumente, Bad Schwalbach, Germany). The used SEM was a “Leica Stereoscan 430i” model.

## 3. Results and Discussion

### 3.1. Deformation Behavior under Torsion

**Figure 5.**
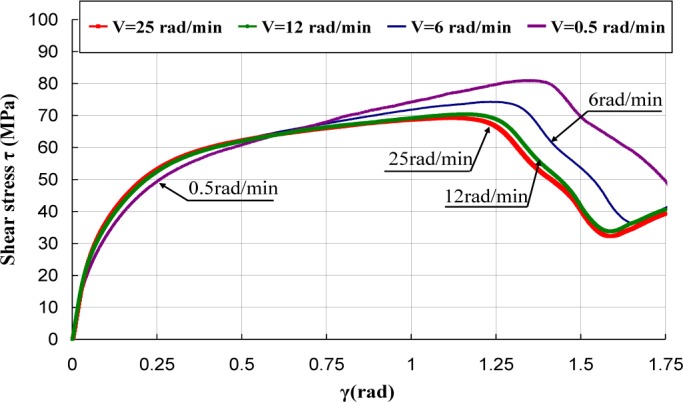
Torsion tests of PA66.

**Figure 6.**
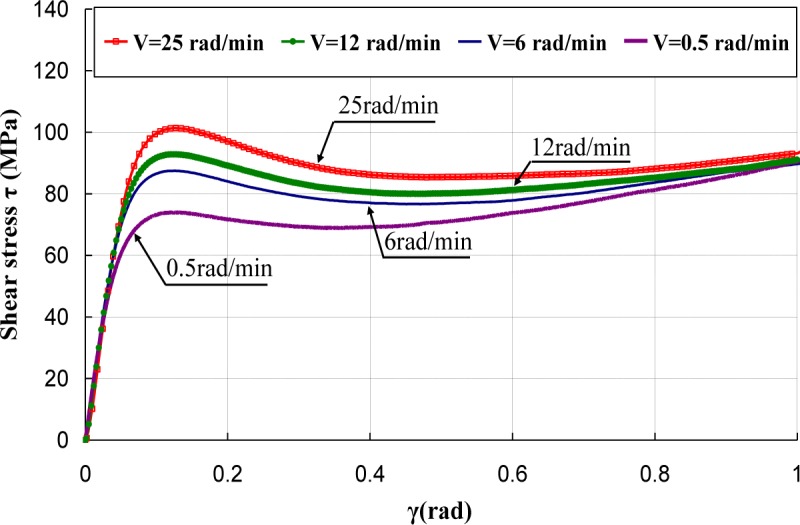
Torsion tests of PMMA.

**Figure 7.**
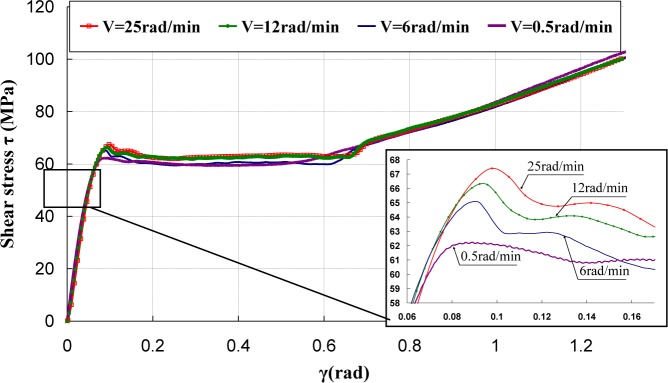
Torsion tests of PC.

Figures 5–7 represent the shear stress during the torsion tests, which were performed at various rotation speeds 0.5, 6, 12, and 25 rad/min, which correspond to the shear strain rates $\dot{\gamma }$ of 3.93 × 10^−3^, 4.71 × 10^−2^, 9.42 × 10^−2^ and of 1.96 × 10^−1^·s^−1^.

It can be observed that the behavior under torsion of these polymers was strongly nonlinear. The analysis of the curves revealed that PA66 samples did not show an intrinsic yield drop when they were subjected to the torsion load, whereas, for PC and PMMA, a peak followed by a drop was observed. Hence, the yield stress τ_0_ was measured by adopting a conventional offset of 0.3% [16,17]. Thus, to determine the experimental yield shear stress τ_0exp_, at which yielding occurred, an equivalent conventional offset of 0.3% was adopted. It is known that the inelastic strain tensor is written as follows [3]:

$$\underline{\underline{\varepsilon }}=\left[\begin{array}{lll} \varepsilon & \frac{\gamma }{2} & 0\\ & \varepsilon & 0\\ & & \varepsilon \end{array}\right]$$ (2)

Hence, the inelastic accumulated equivalent strain is $\varepsilon_{\text{eq}}=\sqrt{2\varepsilon^{2}+\gamma^{2}/3}$.

By neglecting ε, the expression of the equivalent strain ε_eq_ is given by Equation (3):

$$\varepsilon_{\text{eq}}=\frac{\gamma }{\sqrt{3}}$$ (3)

It follows that the adopted angular deviation offset “γ_0_” is equal to 5.2 × 10^−3^ rad. This leads to determine the yield shear stress τ_0exp_ for a given loading condition.

**Figure 8.**
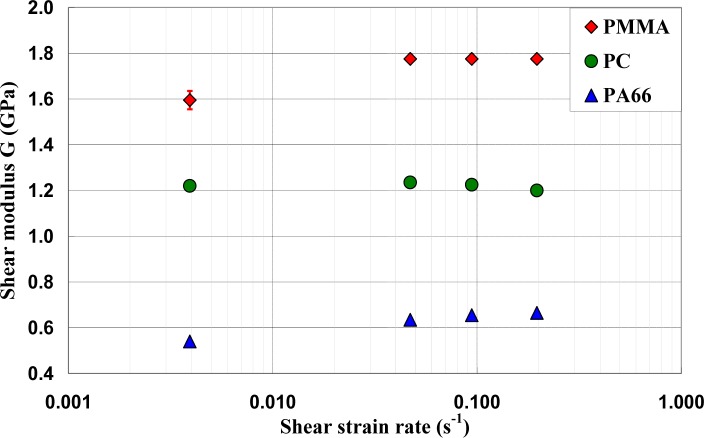
Shear moduli *versus* the equivalent strain rates.

The effect of strain rate was studied at the room temperature. Torsion tests were conducted at different equivalent strain rates ${\dot{\varepsilon }}_{\text{eq}}$ varying in the range of 0.00227 s^−1^ to 0.113 s^−1^. It can be noticed that, for small deformations corresponding to γ ≈ γ_0_, where γ_0_ was the angular deformation required to reach τ_0exp_, the mechanical behavior was quite independent of strain rate, while, as soon as an inelastic angular deviation appeared, the response of the polymers depended strongly on the strain rate. The shear moduli were calculated and their values were reported *versus* the equivalent strain rates ${\dot{\varepsilon }}_{\text{eq}}$ (Figure 8). It can be observed that the shear modulus was independent of strain rate for PC, while a dependency is observed for PA66 and PMMA. However, this dependency is not significant and can be neglected in the constitutive equation of the mechanical behavior of the polymers.

Hence an elasto-visco-plastic constitutive equation was used to describe the mechanical behavior under torsion of these polymers. The elastic region is the result of intermolecular interactions between chains due to van der Waal forces, while plasticity is attributed to molecular movements.

### 3.2. Effect of Strain Rate on the Shear Yield Stress

**Figure 9.**
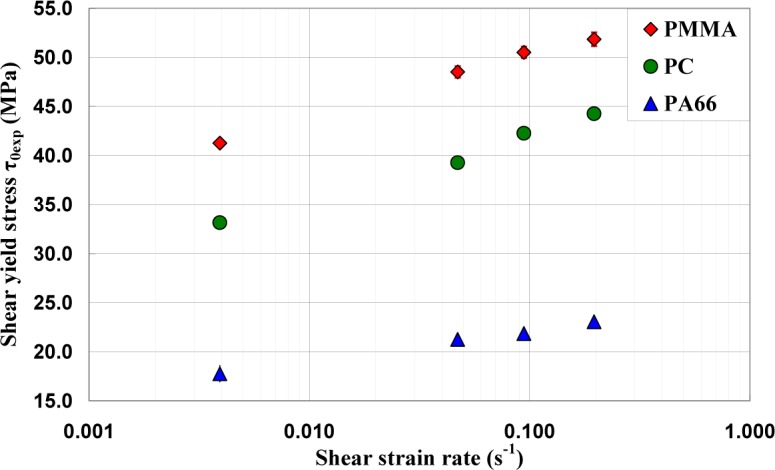
Yield shear stresses τ_0exp_ of PA66, PC, and PMMA as a function of shear strain rate $\dot{\gamma }$.

The values of yield shear stresses τ_0max_ determined by adopting an angular deviation offset γ_0_ equal to 5.2 × 10^−3^ rad (or ε_eq_ = 0.3%) are presented in Figure 9. These values are compared to those determined according to the ISO 6721-6:1996 standard [18] (Table 3). In this last case, the yield shear stress was assumed as the nominal maximum stress reached by the polymer during torsion test. This definition imposed the observation of a load drop. One of the problems in adopting this approach is that the curves obtained under a given loading conditions cannot exhibit the formation of an intrinsic yield drop, such as for PA66 under torsion, while a load drop was observed in the tensile tests.

Several models can be used to fit the rate dependent experimental yield data of polymer behavior. Among these, the Ree-Eyring model, and the more recent Argon model are the most applied ones for amorphous polymers. In this study, both models were used and compared for the prediction of the strain rate effects on yield stress of the polymers subjected to tensile, compressive, and shear tests.

According to Miehe *et al.* [19], the viscoplastic flow of amorphous glassy polymers is a thermally activated, stochastic process. The isotropic resistance to plastic deformation of amorphous glassy polymers is described by the intermolecular barrier to chain segment rotation model originally proposed by Argon and modified by Boyce *et al.* [20]. The rate and temperature dependent flow stress in shear is expressed as follows [21,22]:

$$\tau =(s+\alpha_{p}p)[1+\frac{k_{B}T}{A(s+{\text{α}}_{p}p)}]\text{Ln}{\left(\frac{\dot{\gamma }}{{\dot{\gamma }}_{0}}\right)}^{\frac{5}{6}}$$ (4)

where τ is the rate and temperature dependent shear flow stress, *s* is the shear strength, α*_p_* is the pressure coefficient, *p* is the pressure, *k_B_* is the Boltzmann constant, *T* is the applied absolute temperature, *A*(*s* + α_p_p) is the zero stress level activation energy modified to include pressure dependence, $\dot{\gamma }$ is the applied shear strain rate, and ${\dot{\gamma }}_{0}$ is the pre-exponential factor proportional to the attempt frequency. Thus, the Argon-Boyce equation, which describes the shear yield stress, can be simplified as follows:

$$\frac{\tau_{0\text{exp}}}{T}=a{\left[c-b\text{Ln}\left(\dot{\gamma }\right)\right]}^{\frac{5}{6}}$$ (5)

where *a*, *b*, and *c* are experimental material constants.

From a micromechanical point of view, Equation (4) well-describes polymers’ yield stress dependency on temperature and strain rate. Several researchers adopt this expression, even if the physical arguments underlying Argon’s view are still debated [22].

The model of Eyring, developed in 1936, is widely used to describe the rate-dependent plasticity in amorphous polymers. In Eyring’s theory, yield processes are thermally activated and molecular motions can be carried on only if an energy barrier is overcome. This model indicates that yielding in polymers is controlled by cooperative movements of several molecular chains. Hence, the representation of the yield stress *versus* logarithm of the strain rate should be linear for a given temperature (assumption of a single thermal activation mechanism). It is the case of the polycarbonate yield behavior for a wide field of temperatures and strain rates [23].

However, neither Eyring nor Argon models were applied as much as the Ree-Eyring model, which was established in 1955, to fit the rate dependent yield data of amorphous polymers. This model, based on several activation processes [24], is a modification of the Eryring model and its expression is given by Equation (6) where *a*, *b*, and *c* are materials constants.

$$\frac{\tau_{0\text{exp}}}{T}=a\text{Ln}\left({\dot{\varepsilon }}_{\text{eq}}\right)+b{\sin\text{h}}^{-1}\left(d{\dot{\varepsilon }}_{\text{eq}}\right)+c.\;\text{Where}\;{\dot{\varepsilon }}_{\text{eq}}=\frac{\dot{\gamma }}{\sqrt{3}}$$ (6)

**Figure 10.**
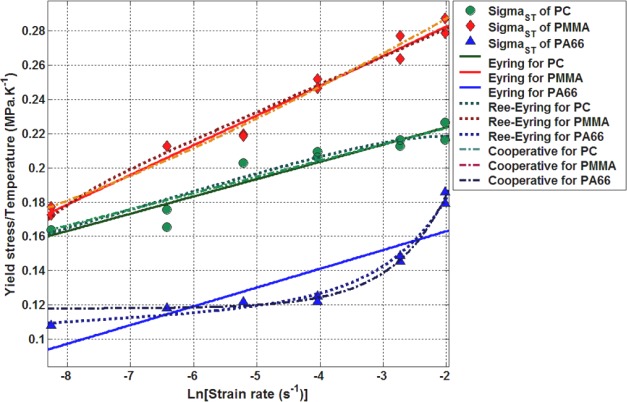
Evolution of the yield stress as a function of logarithm of strain rate [25].

**Figure 11.**
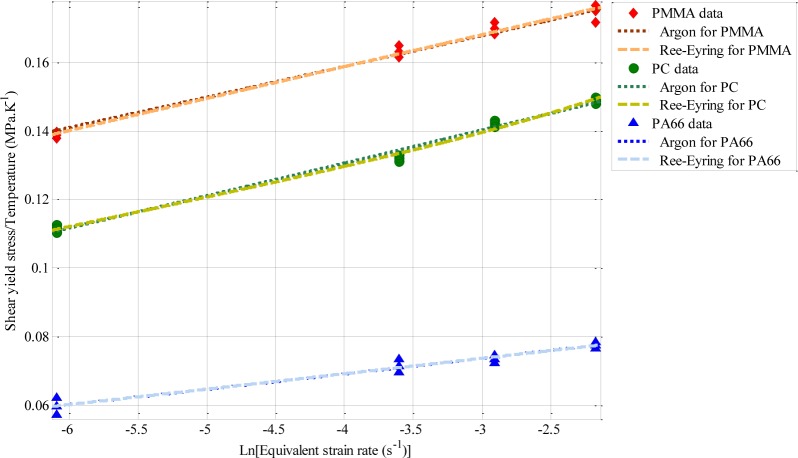
Best-fit evolution of the shear yield stress as a function of logarithm of equivalent strain rate [25].

The tensile deformation behavior of the same polymers was studied by the authors in a previous work [25]. It was established that the best model predicting tensile yield stress, σ_st_ of amorphous and semi-crystalline polymers is the Ree-Eyring model (Figure 10). Moreover, it appears that both Argon model and Ree-Eyring predict successfully the tensile yield stress σ_st_ at moderate strain rates (Figure 11).

In this study both models were used to model the effect of strain rate on the shear yield stress of PC, PA66, and PMMA.

Argon and Ree-Eyring models were used to fit the experimental data. The curves parameters are summarized in Table 4. It appears that Ree-Eyring model provided the best prediction of shear yield stress. Hence, it can be suitable to introduce the Ree-Eyring model in the constitutive equations dedicated to the modeling of the thermo-mechanical behavior of glassy and semi-crystalline polymers.

### 3.3. The Yield Criterion

During the torsion test the polymers showed a linear elastic behavior until the yield point had been reached. Then, the viscoplasticity took place. Several models, based on thermodynamics with internal variables, can be used to predict the thermo-mechanical behavior of polymers under service conditions. The method is based on the definition of a yield function to express the form of the viscoplastic potential and to establish the equations governing hardening variables. Some yield criteria, based on the classical plasticity, including hydrostatic pressure effects and assumptions, such as isotropy, were previously established and widely used, such as those derived from the modified Von Mises criteria (MMC) and the Drucker–Prager yield criterion (MMP). They are expressed in the normalized plane by the equations Equation (7) and are based on two stresses invariants *I*_1_ and *J*_2_.

$$\begin{array}{l} {\left(\frac{\sqrt{3J_{2}}}{\sigma_{\text{ST}}}\right)}^{n}+\frac{m^{n}-1}{m+1}\left(\frac{I_{1}}{\sigma_{\text{ST}}}\right)=\frac{m(m^{n-1}+a)}{m+1},\text{where }m=\frac{\sigma_{\text{SC}}}{\sigma_{\text{ST}}}\geq \text{1 and }\{\begin{array}{c} n=1\;\Rightarrow \text{ MMC}\\ n=2\;\Rightarrow \text{ MMP} \end{array}\\ \text{where }I_{\text{1}}=tr\left(\underline{\underline{\sigma }}\right)\text{ , }J_{\text{2}}=\frac{1}{2}\cdot tr({\underline{\underline{S}}}^{2}\text{) and }\underline{\underline{S}}=\underline{\underline{\sigma }}-\frac{tr\left(\underline{\underline{\sigma }}\right)}{3}\underline{\underline{I}} \end{array}$$ (7)

where σ_ST_ is the tensile yield stress and σ_SC_ is the uniaxial compressive yield stress.

**Figure 12.**
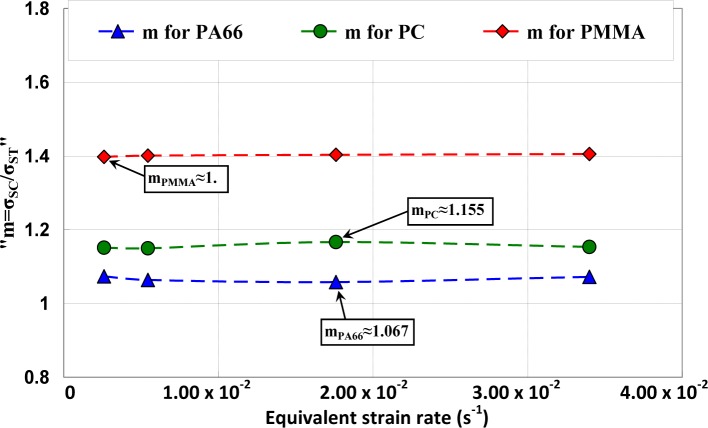
Relationship between the tensile yield stress and the uniaxial compressive yield stress as a function of equivalent strain rate.

As it was established, yield stress was dependent on temperature and strain rate and pressure effects. The compressive and tensile tests were performed on the studied polymers to investigate the effect of the equivalent strain rate on the parameter “ $m=\frac{\sigma_{\text{SC}}}{\sigma_{\text{ST}}}$”. The obtained results are presented in Figure 12 where it clearly appears that “*m*” is constant as a function of equivalent strain rate and is estimated equal to 1.4 for PMMA, equal to 1.155 for PC, and equal to 1.067 for PA66.

Both criteria well described the polymers yielding behavior for high hydrostatic pressure values. However, they did not allow an accurate description when the main deformation mechanism occurred by shear banding. In fact, both of them were derived from the von Mises model, based on a non-directional octahedral shear stress more appropriate for the description of homogeneous deformation mechanisms. To improve the prediction of isotropic polymers yielding behavior a generalized yield criterion was proposed [1]. Its expression is given by Equation (8), based on three stress invariants, *I*_1_, *J*_2_, and *J*_3_.

$$\begin{array}{l} f=\frac{3J_{2}}{\sigma_{\text{ST}}}\psi (J_{2},J_{3})+(m+1)\left(1-\frac{\kappa }{8}\right)I_{1}-\left(1-\frac{\kappa }{8}\right)m{\text{ σ}}_{\text{ST}},\\ \text{where }J_{3}=\frac{1}{3}\cdot tr\left({\underline{\underline{S}}}^{3}\right),\psi \left(J_{2}\text{,}J_{3}\right)=\left(1-\frac{27}{32}\kappa \frac{J_{3}^{2}}{J_{2}^{3}}\right),\text{ κ}=\left(1-\frac{3r}{m}\right)\text{ and }r=\frac{\tau_{\text{0exp}}}{\sigma_{\text{ST}}} \end{array}$$ (8)

where τ_0exp_ is the yield stress determined from simple shear test. It can be observed that when κ_0_ = 0 (*i.e.*, $r=\sqrt{m/3}$), the dependence on the third deviatoric stress invariant is removed; the MMP criterion is recovered. The convexity of the yield surface is ensured for 0 ≤ κ ≤ 1.

**Figure 13.**
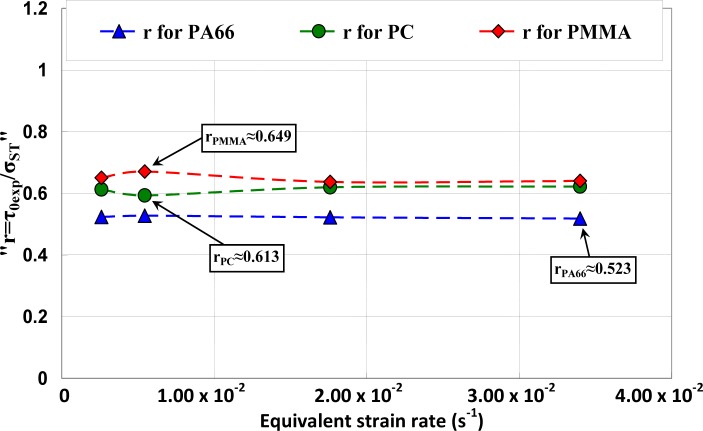
Relationship between yield stress and yield shear stress as a function of equivalent strain rate.

The ratio r was calculated and its evolution as a function of the strain rate was investigated. It can be observed (Figure 13) that *r* was quasi-constant for a given polymer.

To reduce the number of tests necessary to the identification of the parameters of the suggested criterion the authors proposed a simplification by setting κ = 1, which did not affect the convexity of the function *f*. The assumption of κ = 1 meant that $r=\sqrt{m/3}$ and therefore $\frac{3\cdot r^{2}}{m}$ could be neglected with regard to 1. In other words, the yield stress obtained experimentally through shear tests was lower than the predicted one using the yield function: $\tau_{o}=\sqrt{\frac{m}{3}}\sigma \text{ST}$. Hence, Equation (8) was reduced to Equation (9) which represents the expression of the PROPOSED yield function in this study.

$$f=\frac{3\cdot J_{2}}{\sigma_{\text{ST}}}\cdot \Psi \left(J_{2},J_{3}\right)+\frac{7.\left(m-1\right)}{8}\cdot I_{1}-\frac{7\cdot m}{8}\cdot \sigma_{\text{ST}}\text{ with }\Psi \left(J_{2},J_{3}\right)=\left(1-\frac{27}{32}\cdot \frac{J_{3}^{2}}{J_{2}^{3}}\right)$$ (9)

**Figure 14.**
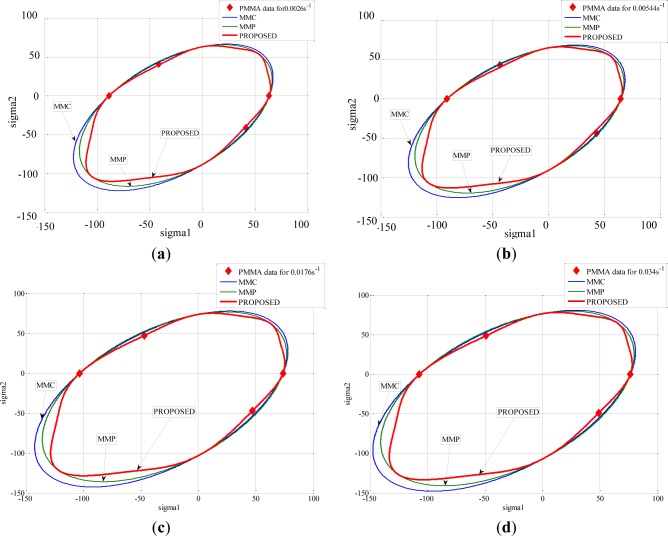
Predicted yield stress using different criteria and experimental results of PMMA represented in the principal stress plane (σ_I_ *versus* σ_II_): for equivalent strain rate (**a**) 0.0026 s^−1^; (**b**) 0.00544 s^−1^; (**c**) 0.0176 s^−1^; and(**d**) 0.0034 s^−1^.

**Figure 15.**
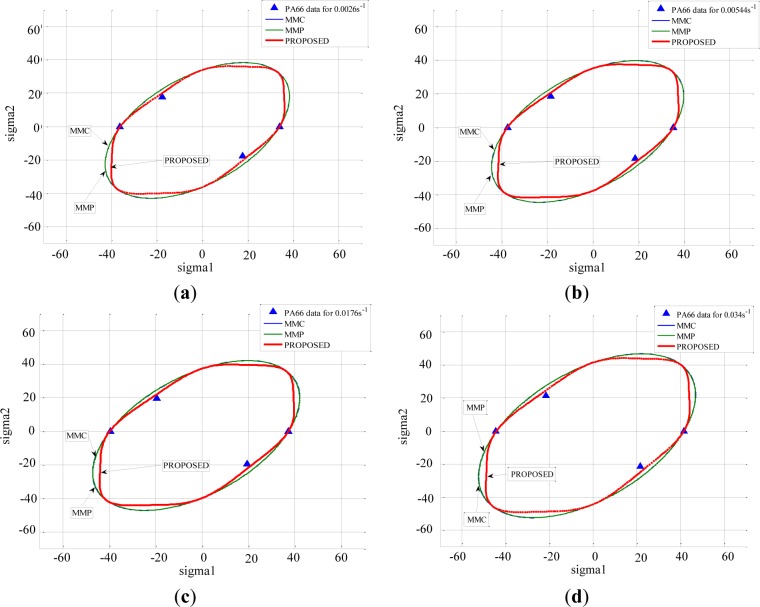
Predicted yield stress using different criteria and experimental results of PA66 represented in the principal stress plane (σ_I_ *versus* σ_II_): for equivalent strain rate (**a**) 0.0026 s^−1^; (**b**) 0.00544 s^−1^; (**c**) 0.0176 s^−1^; and (**d**) 0.0034 s^−1^.

The different criteria (MMP, MMC, and PROPOSED) were compared, and their ability to describe the yielding of these polymers was discussed. It was observed that the introduction of the third invariant did not affect the yield strength in the uniaxial tension load, in uniaxial compression load and under hydrostatic pressure condition. This was in agreement with the deformation mechanisms taking place for those loading conditions, which did not involve rotation. Moreover, it was observed that the PROPOSED criterion allowed matching the experimental results better than the other criteria when polymers were subjected to shear tests at high strain rates for PMMA (Figure 14) and for PA66 (Figure 15). The explanation lies in the fact that the main deformation mechanism for semi-crystalline polymers occurred mainly by shear banding.

**Figure 16.**
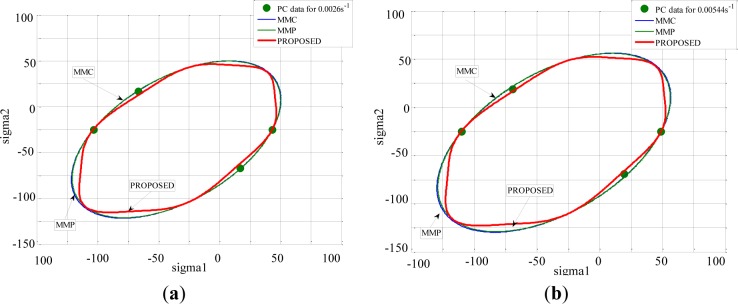

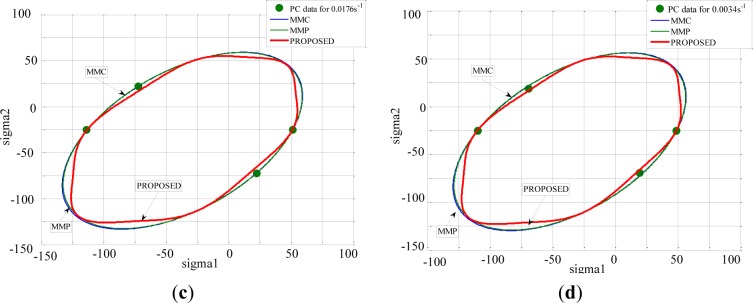
Predicted yield stress using different criteria and experimental results of PC represented in the principal stress plane (σ_I_ *versus* σ_II_): for equivalent strain rate (**a**) 0.0026 s^−1^; (**b**) 0.00544 s^−1^; (**c**) 0.0176 s^−1^; and (**d**) 0.0034 s^−1^.

For PC, which deformed non-homogeneously, no differences could be observed between the three yield surfaces (Figure 16). Despite the improvements provided by modifying the yield function, some differences were observed between predicted and experimental values of the shear stress at yielding for the semi-crystalline polymer PA66, which were slightly over-estimated.

To conclude, the PROPOSED yield function allowed predicting correctly the yielding of amorphous and semi-crystalline polymers for a wide range of strain rates. The dependency of the yield stress on temperature and strain rate was well-reproduced by the Ree-Eyring model.

### 3.4. Self-Heating Induced by Torsion Deformation

**Figure 17.**
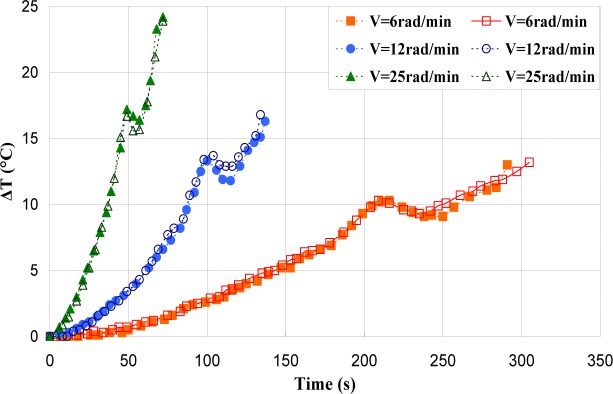
Self-heating of PA66 induced by torsion deformation.

**Figure 18.**
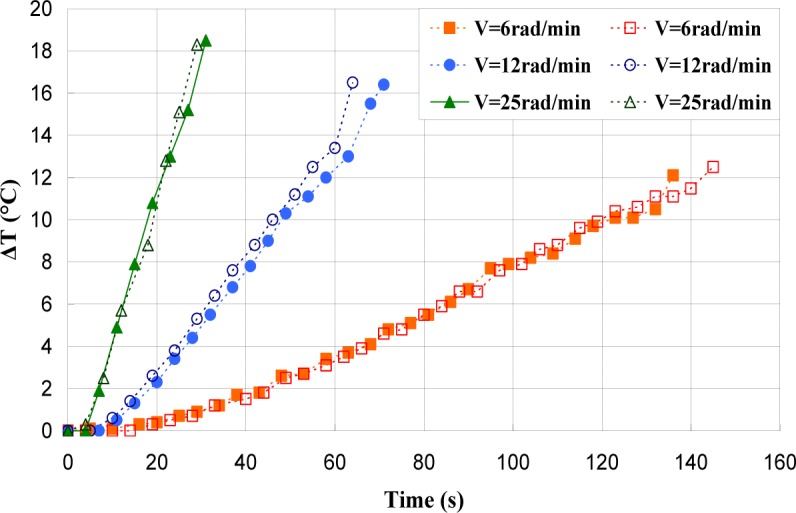
Self-heating of PMMA induced by torsion deformation.

**Figure 19.**
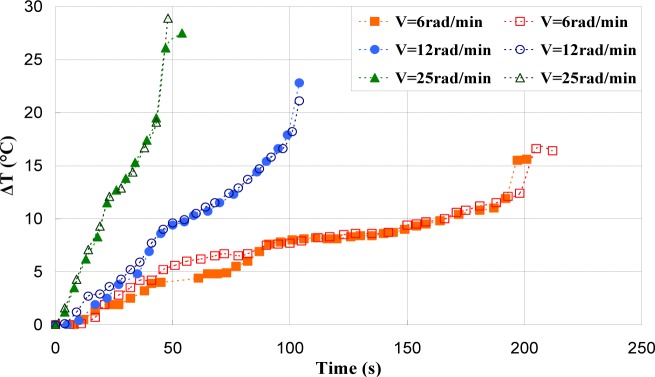
Self-heating of PC induced by torsion deformation.

Figures 17–19 show the evolution of the maximum temperature recorded at the specimen surface during the deformation of PA66, PMMA, and PC, respectively. It indicates that, as the strain rate was increased the surface temperature increased due to the viscoplastic energy. On the other hand, no rise in temperature was detected for a loaded deformation of 0.5 rad/min corresponding to a strain rate equal to 2.27 × 10^−3^·s^−1^. Moreover, a rise in the temperature above 28 °C in PC was noticed for a 25 rad/min rate strain. It is interesting to note that this phenomenon was more significant when the strain rate increased. The assumption that those tests were isothermal was debated.

Hence, as soon as the polymer was inelastically strained, the temperature increased and the polymer softened. The observed yield drop corresponded to the contribution of the intrinsic strain and thermal softening of the polymer. With large deformations, competing effects occurred and thermal softening offset the strain hardening.

For the same experimental condition (*V =* 25 rad/min), the temperature rise depends on the polymer.

**Figure 20.**
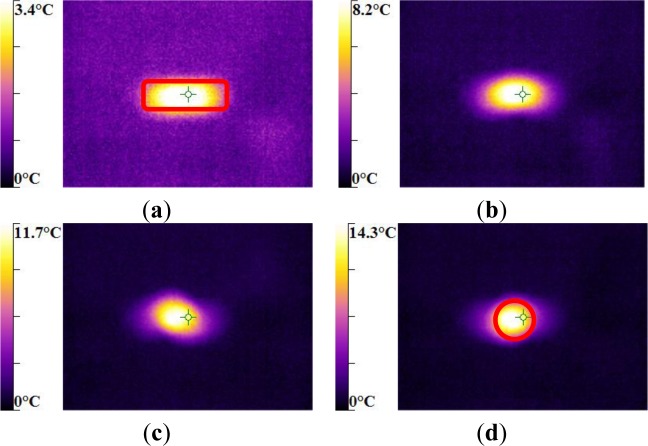
IR Thermographs of PA66 torsion test for *V =* 12 rad/min. (**a**) γ = 0.73 rad; (**b**) γ = 1.18 rad; (**c**) γ = 1.39 rad; and (**d**) γ = 1.86 rad.

**Figure 21.**
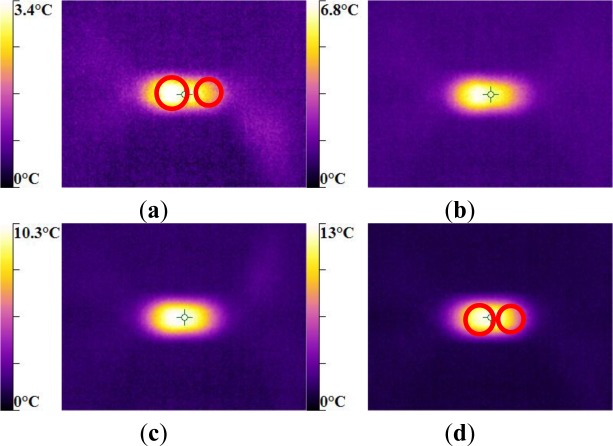
IR Thermographs of PMMA torsion test for *V =* 12 rad/min. (**a**) γ = 0.36 rad; (**b**) γ = 0.56 rad; (**c**) γ = 0.74 rad; and (**d**) γ = 0.94 rad.

**Figure 22.**
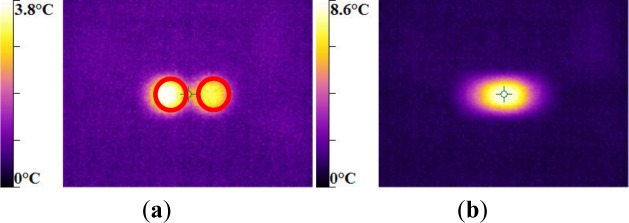

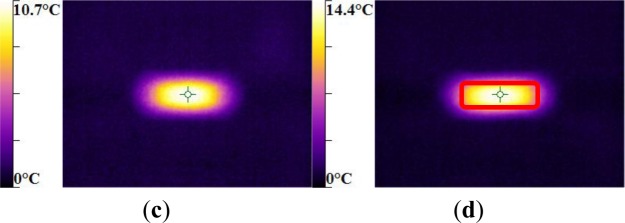
IR Thermographs of PC torsion test for *V* = 12 rad/min. **a**) γ = 0.41 rad; (**b**) γ = 0.68 rad; (**c**) γ = 0.97 rad; and (**d**) γ = 1.29 rad.

**Figure 23.**
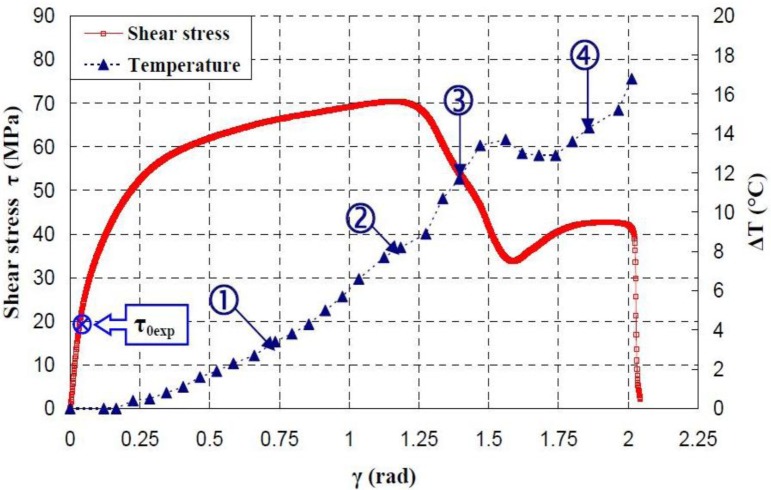
Thermal dissipation and shear stress of PA66 polymers during the torsion test.

**Figure 24.**
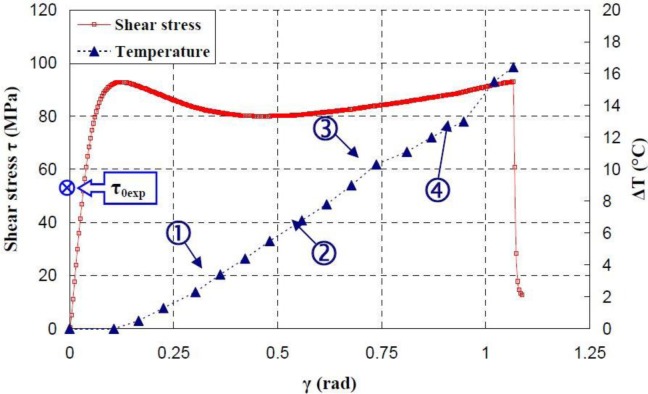
Thermal dissipation and shear stress of PMMA polymers during the torsion test.

**Figure 25.**
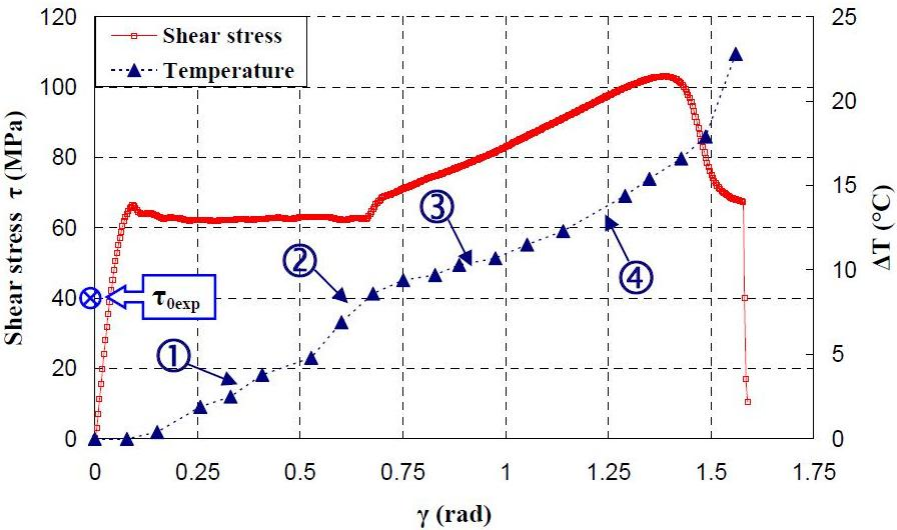
Thermal dissipation and shear stress of PC polymers during the torsion test.

The maximum was roughly 18.5 °C for the PMMA, 24 °C for the PA66, while it exceeded 28 °C in the case of the PC. From the thermography analysis (Figures 20–22), it appeared that the temperature distribution was not uniform throughout the surface of the samples. Hence, it can be assumed reasonably that the deformation mechanisms were not homogeneous, especially after yielding. These results proved the correctness of introducing the third invariant in the yield surface to take into account all deformation mechanisms occurring under different mechanical loading conditions. The stress-strain response was plotted *versus* the heat temperature measurements (Figures 23–25).

For the PA66 semi-crystalline polymer, the heat distribution was extended to all surfaces as the stress exceeds the yield stress (Figure 23).

This was not the same in the case of the amorphous polymers. After reaching the yield stress, two points of heat source were located on the specimen extremities of PC and PMMA (Figures 24 and 25). For PMMA, as the deformation increased, these sources extended and merged together until the fracture occurred. These results showed, qualitatively, that self-heating was linked to the strain rate on the one hand and to the type of polymer (according to its *Tg* and degree of crystallinity) on the other hand.

The self-heating dissipation after reaching the yield stress during the torsional test can be related to the deformation mechanisms of polymers during the material flow. It has to be introduced into the constitutive equations dedicated to model the mechanical behavior of polymers.

### 3.5. SEM Fractography

The effect of strain rates on failure modes of polymers subjected to torsion was analyzed by scanning electron microscopy. Fracture involved breaking of covalent bonds in the chains and exhibited characteristic features, such as a mirror zone at the origin, a mist region, and rib marks. The investigated polymers showed either brittle or ductile fracture behaviors [26].

**Figure 26.**
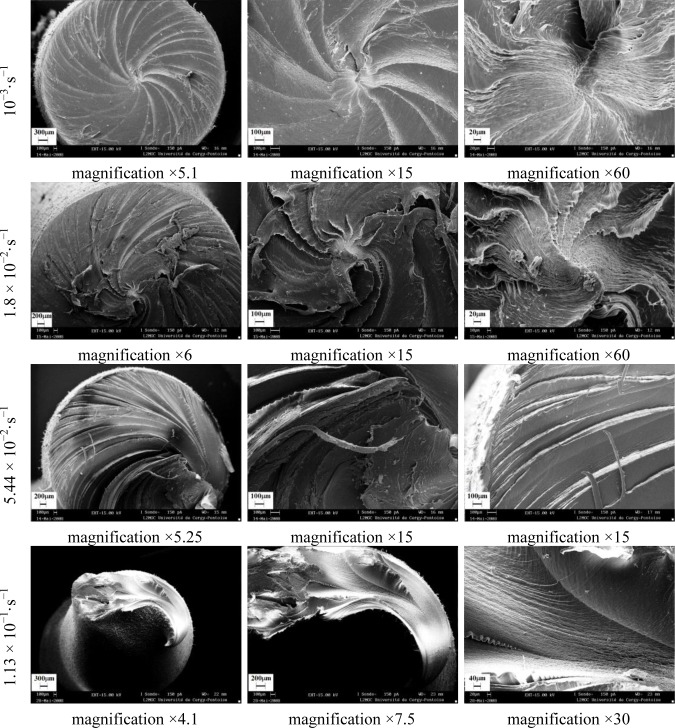
Fractography of PA66 torsion test for different equivalent strain rates.

**Figure 27.**
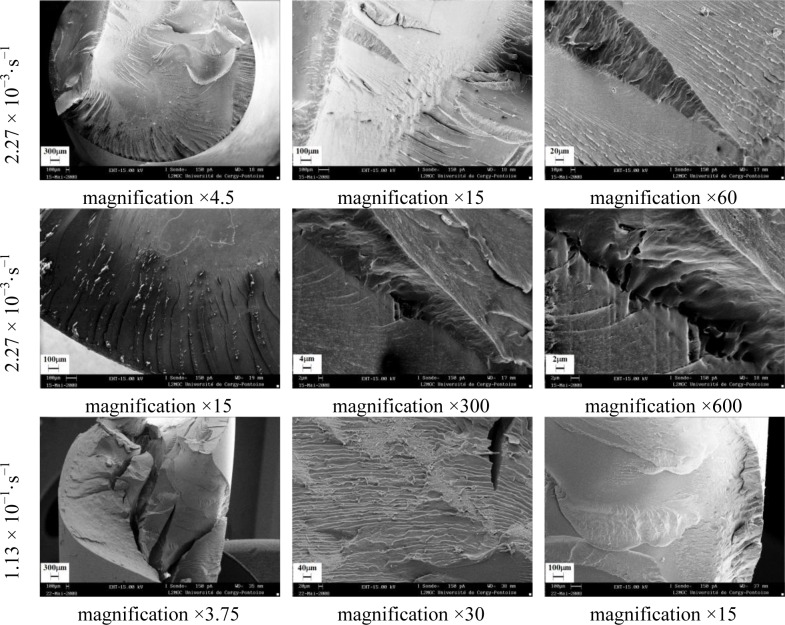
Fractography of PMMA torsion test for different equivalent strain rates.

**Figure 28.**
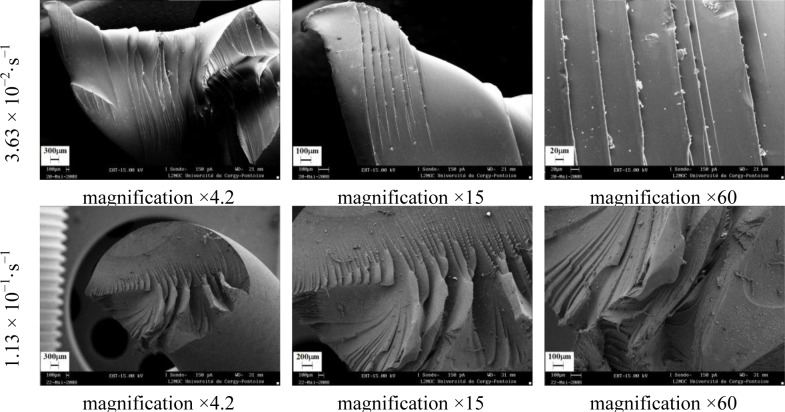

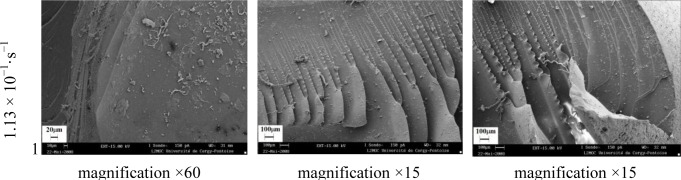
Fractography of PC torsion test for different equivalent strain rates.

Figures 26–28 show the collected SEM images for PA66, PMMA, and PC. These images give an account, at a glance, of the complex propagation of the fracture surfaces for different strain rates.

It can be seen that the torsional shear stress produced the typical striations pattern. A change in the fracture pattern was typically observed for equivalent strain rates higher than 1.8 × 10^−2^·s^−1^ for PA66 (Figure 26). The fracture surface of the failed specimens revealed ductile zones with peak formation. Ductile failure was slow and used a great deal of energy in the failure. Cracks grew slowly, the extension (strain) at final fracture was high, and the fracture surfaces tended to have a fibrous appearance with a peaks length >10 μm The transition can be attributed to the self-heating, which increased with higher strain rates. By increasing the strain rate, the temperature increased, which resulted in an important softening of the polymer and, therefore, a change in failure mode from brittle to ductile. The transition from ductile to brittle behavior was also affected by the speed of crack growth in the material.

During the brittle fracture, the cleavage stopped and the fracture plans recombined during the course of further propagation. Chips formed in front of the crack propagation line and they explain the occurrence of step-like cracking patterns. The peaks length was less than 1 μm. PMMA and PC showed brittle behavior, which was not significantly influenced by the strain rate (Figures 27 and 28). The PMMA susceptibility to cracking and brittle fracture was also found during compression testing. It was due to any local inhomogeneity in the specimen that could lead to a local tensile stress, which initialized the brittle failure [27].

## 4. Conclusions

The investigation concerned the effects of the strain rate on the mechanical behavior of thermoplastics polymers. It was demonstrated that a third invariant had to be introduced in order to predict correctly the polymers yielding behavior. A yield function was proposed and the dependency of the yield stress on the temperature and strain rate was taken into account using the Ree-Eyring model.

Self-heating dissipation occurred after reaching the yield stress for all polymers during the torsion test. It was related to both the deformation mechanism and the strain rate.

The strain rate yielding criterion described the self-heating effects on the mechanical and the fractures behavior of the investigated thermoplastics polymers, which provided an opportunity to explain failure process and to illustrate the loading limit of the materials.

Hence, as soon as the polymer was inelastically strained, the temperature increased and the polymer softened. The yield drop, which was observed when the polymer was loaded, corresponded to the contribution of the intrinsic strain and the thermal softening. The strain hardening balanced the thermal softening effect when large deformations occurred.

The description and classification of the fracture mode were realized by SEM analysis.

PC and PMMA exhibited brittle fracture, with a mirror zone at the origin, a mist region, and rib marks. The brittle fracture was insensitive to the equivalent strain rate.

Brittle-ductile transition was observed for PA66 at an equivalent strain rate higher than 1.8 × 10^−2^·s^−1^, which made PA66 fracture behavior more temperature-sensitive than that of PMMA and PC.

## Figures and Tables

**Table 1.** The assignment of principal infrared bands of polymethyl methacrylate (PMMA), polyamide (PA66), and polycarbonate (PC).

Polymer	NO.	Wavenumber (cm^−1^)	Peak assignments
PMMA	❶	2360–2952	O–CH_3_ stretch
	❷	1727	C=O stretch (Ketone)
	❸	1450	C–H bending (Alkane)
	❹	1149	C–O stretch (Ether)
	❺	751–988	(CH_2_)*_n_* with *n* ≥ 4 (Alkane)
PA66	❶	3292	N–H stretch (Amide)
	❷	1641	C=O stretch (Ketone)
	❸	1198	C–N stretch (Amine)
	❹	728–935	(CH_2_)*_n_* with *n* ≥ 4 (Alkane)
PC	❶	2972	C–H stretch (Aromatic)
	❷	1780	C=O stretch (Ketone)
	❸	1501	C=C stretch (Aromatic)
	❹	1189	C–O stretch (Ether)
	❺	1004	C–O stretch (Ether)
	❻	554	(CH_2_)*_n_* with *n* ≥ 4 (Alkane)

**Table 2.** Thermal properties of studied polymers [15].

Thermal properties	PC	PMMA	PA66
*T_g_* (°C)	149.7	127.7	58.8
*T_f_* (°C)	*	*	261.4
Δ*H_f_* (J/g)	*	*	47.7
*X_c_* (%)	*	*	25

* No *T_f_* for amorphous polymers.

**Table 3.** Yield shear stresses τ_0max_.

V (rad/min)	Shear strain rate (s^−1^)	PC		PA66		PMMA	

		τ_0max_ (MPa)	s **	τ_0max_ (MPa)	s **	τ_0max_ (MPa)	s **
0.5	3.93 × 10^−3^	61.8	0.35	*	*	74.1	0.3
6	4.71 × 10^−2^	64.8	0.23	*	*	87.8	0.73
12	9.42 × 10^−2^	66.1	0.17	*	*	92.7	0.1
25	1.96 × 10^−1^	67.3	0.67	*	*	100.8	0.83

* ISO R257 standard can’t be applied; ** Standard deviation.

**Table 4.** Best-fit parameters for Argon’s and Ree-Eyring’s model.

Material	Model’s	Equation model	*a*	*b*	*c*	*d*	*R*^2^
PMMA	Argon	$\frac{\tau_{0\text{exp}}}{T}=a{\left[c-\text{Ln}({\dot{\varepsilon }}_{\text{eq}})\right]}^{\frac{5}{6}}$	5.7 × 10^−6^	−239.1	6023	–	0.9819
	Ree-Eyring	$\frac{\tau_{0\text{exp}}}{T}=a\text{Ln}({\dot{\text{ε}}}_{\text{eq}})+b{\sin\text{h}}^{-1}(d{\dot{\varepsilon }}_{\text{eq}})+c$	3.632 × 10^−2^	−2.7 × 10^−2^	0.4362	3623	0.9840
PC	Argon	$\frac{\tau_{0\text{exp}}}{T}=a{\left[c-b\text{Ln}({\dot{\varepsilon }}_{\text{eq}})\right]}^{\frac{5}{6}}$	9.8 × 10^−5^	−24.55	499.8	–	0.9877
	Ree-Eyring	$\frac{\tau_{0\text{exp}}}{T}=a\text{Ln}({\dot{\varepsilon }}_{\text{eq}})+b{\sin\text{h}}^{-1}(d{\dot{\varepsilon }}_{\text{eq}})+c$	8.4 × 10^−3^	−1.414 × 10^−2^	0.1625	−3.34	0.9911
PA66	Argon	$\frac{\tau_{0\text{exp}}}{T}=a{\left[c-b\text{Ln}({\dot{\varepsilon }}_{\text{eq}})\right]}^{\frac{5}{6}}$	2.396 × 10^−3^	−0.8966	20.07	–	0.9532
	Ree-Eyring	$\frac{\tau_{0\text{exp}}}{T}=a\text{Ln}({\dot{\varepsilon }}_{\text{eq}})+b{\sin\text{h}}^{-1}(d{\dot{\varepsilon }}_{\text{eq}})+c$	4.49 × 10^−3^	−58.1	8.716 × 10^−2^	−4.819 × 10^−3^	0.9544
